# Insight towards Nucleation Mechanism and Change in Morphology for Nanostructured Platinum Thin Film Directly Grown on Carbon Substrate via Electrochemical Deposition

**DOI:** 10.3390/ma14092330

**Published:** 2021-04-30

**Authors:** Prabhakaran Dhanasekaran, Swaminathan Rajavarman, Sivasuriyanarayanan Vinod Selvaganesh, Santoshkumar Dattatray Bhat

**Affiliations:** 1 CSIR-Central Electrochemical Research Institute (CECRI), CSIR-Madras Complex, Tamil Nadu 600113, India; 2 Centre for Nanoscience and Nanotechnology, University of Madras, Tamil Nadu 600085, India; srajavarman11@gmail.com; 3 Indian Institute of Technology, Madras, Chennai, Tamil Nadu 600036, India; svsganesh.dr@gmail.com

**Keywords:** different morphology, electrochemical deposition, fuel cell, durability, PEFC

## Abstract

Nanocrystalline platinum with different morphologies is synthesized via electrochemical deposition technique. The nucleation mechanism and its structural effect over the electrodeposited Pt on carbon electrodes have been systematically studied. Powder X-ray diffraction, X-ray photoelectron spectroscopy, and field-emission scanning electron microscopy are employed to study nucleation, oxidation states, and Pt structure growth on a carbon electrode. This study reports significant development of Pt metal nanoparticles with different morphologies such as a sphere, flower, core-flower, and rod-like structure directly fabricated on carbon electrode while tuning the deposition parameters such as current density, time, temperature, pH during the deposition process. The proposed electrochemical route represents a superior fabrication procedure for large-scale electrode fabrication compared to a conventional method for preparing membrane electrode assemblies for fuel cell stacks.

## 1. Introduction

Fabrication and surface engineering of platinum metal nanoparticles with different morphology and crystal-face has been the focus of many recent studies due to the broad class and most promising applications such as polymer electrolyte fuel cell [1,2], industrial synthesis of ammonia [3], electrochemical oxidation of ethanol and methanol [4,5,6], glucose detection [7], carbon monoxide gas sensors [8], petroleum refining [9], hydrogen production and anticancer drugs [10,11]. The preparation of metal particles with well-controlled size and shapes are more important for catalytic applications. For various catalytic applications, the rate per unit of the surface area of metal is customarily dependent on the crystalline size, morphology, and then crystalline plane orientation generally referred to as structure-sensitive responses [12]. In the past decade, various wet chemical processes, such as hydrothermal, sol-gel, microwave, polyol, colloidal, sodium borohydride reduction, impregnation, etc., have been employed for synthesizing nanocrystalline as well as polycrystalline metal nanoparticles [13,14,15].

In the current scenario, these methods have also offered control over the morphology and particle size while varying different organic polymer surfactants and reducing agents. In the last decade, platinum metal nanoparticles appear to be one of the most efficient catalysts for various applications. There are fewer reports available to synthesize controlled-size and shapes of nanoparticles using adequate polymers [16]. Ramirez et al. reported that various shaped Pt nanostructures such as dendrites or nanowires and isolated nanoparticles were obtained using Tris(dibenzylideneacetone) diplatinum used as a precursor [17]. Similarly, our previous work also reported that Pt metal nanoparticle with a size of 3–5 nm was realized by a simple ethylene glycol reduction method [18].

Although a wide diversity of methodologies to prepare Pt metal particles has been explored, methods that can preserve the shape and size of Pt metal in similar materials have been scanty. In view of this, in the present study, different Pt metal particle morphology is explored via electrochemical deposition. Besides, through the electrochemical deposition technique, one can control the purity, crystallinity, particle size, and nucleation, as well as deposition amount and surface morphology, while altering various deposition parameters. In a typical electrochemical deposition process, metal ion precursors are electrochemically reduced to their metallic state by direct electron transfer from the working electrode [19,20,21].

Liu et al. prepared a cubic-shaped Pt nanoparticle by a one-step electrochemical method with the formulated electrolyte of chloroplatinic acid and hydrochloric acid [22]. Y. Song et al. reported that Pd dendritic nanowires were directly deposited onto ITO glass with the optimum electrolyte of H_3_BO_3_ and PdSO_4_ at room temperature [23]. Jia et al. prepared uniform nanoplates and nano-trees of Pd through electrodeposition on a gold substrate by a template-free method [24]. Similarly, Takai et al. obtained a Pt film with three different mesostructures, such as two-dimensional-hexagonal, cage-type, and globular shape synthesized by adjusting the composition ratio between block co-polymer (polystyrene-b-polyethene oxide) and Pt ion precursor solutions [25]. Moreover, many capping agents are introduced, which results in the formation of heterogeneous impurities and requires post-treatment to remove them considering the chemical preparation route. However, in the case of electrochemical deposition of Pt, the metal nanoparticles could be directly grown on a conductive substrate or the electrode for various electrochemical applications.

**Scheme 1:**
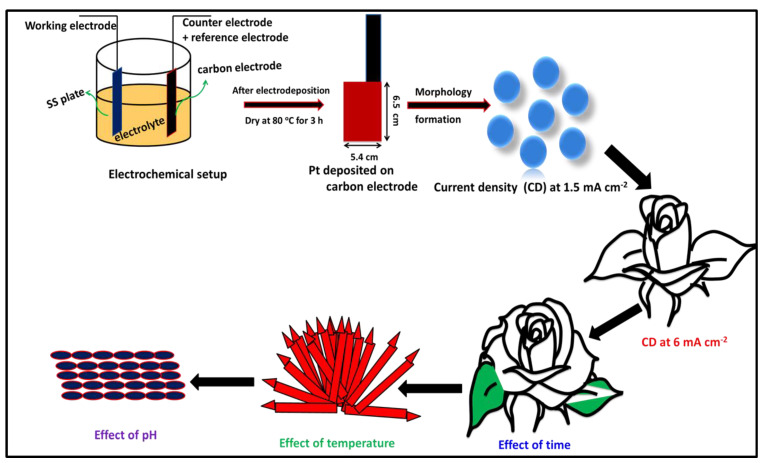
Schematic diagram of electrochemical two electrode systems and formulated electrolyte with exposed conductive carbon substrate (5.4 cm × 6.5 cm) and proposed Pt morphology.

One-step synthesis of Pt nanoparticles of various morphologies via electrochemical deposition was performed on a conductive carbon substrate in the present study. The overall synthesis process is simple, wherein only chloroplatinic acid, citric acid, and sulfuric acid are required. As far as we are aware, this is the first-ever report on the simultaneous direct fabrication of sphere, flower-, and core-flower-shaped Pt metal with (111) facets orientation on a larger active area substrate of 35 cm^2^ by electrochemical deposition. Also, the effect of temperature, time, and pH have been significantly explored. The present work addresses both morphologies as well as facets of orientation. The successful formation of Pt metal nanosphere, flower, core-flower, and rod fenced with (111) planes are confirmed by field emission scanning electron microscopy (FE-SEM), powder X-ray diffraction (XRD), and high-resolution transmission electron microscopy (HR-TEM). Its application in fuel cells as the electrode is also studied. Scheme 1 illustrates Pt metal nanostructure directly deposited on carbon substrate using one-step electrochemical deposition technique and its expected surface morphology while varying current density, time, temperature, and pH.

## 2. Experimental Section

### 2.1. Electrodeposition of Pt Metal Nanoparticles

An electrochemical workstation system (Autolab PGSTAT-30, Ecochemie—Metrohm, The Netherlands) was used for the experiments, wherein the Pt metal nanoparticles were electrodeposited on a carbon substrate. Stainless steel (316 L) with an active area of 80 cm^2^ was used as a working electrode, and a carbon paper (commercial DC-35 SIGRACET^®^ Fuel Cell components (Wiesbaden, Germany) with a thickness of 0.300 mm and an active area of 35 cm^2^) acted as a counter electrode for electrodeposition experiments. During the experiment, the rest of the carbon paper and backside of the active area was covered with Teflon tape. In the present work, the reference electrode was inter-connected with the counter electrode to form a two-electrode system. Before the electrodeposition process, both electrodes were separately immersed in diluted HCl for 5 min, followed by frequent rinsing with adequate distilled water. The plating bath composition comprises exactly 1 mM of H_2_PtCl_6_, 13 g of citric acid, and 0.25 M H_2_SO_4_ and the overall electrolyte adjusted to 500 mL. The platinum nanoparticle was effectively deposited on carbon substrate at various current densities such as 1.5, 3, 6, 9, and 15 mA cm^−2^ for 600 s. Prior to Pt deposition, the carbon paper was electrochemically activated by applying −5 mA cm^−2^ for 20 s in 0.5 M of H_2_SO_4_. Finally, the deposited substrate material was repeatedly rinsed with distilled water, followed by drying at 80 °C for 3 h.

### 2.2. Materials Characterization

Elemental composition and surface morphology were examined by field emission scanning electron microscopy (FE-SEM) on a TESCAN MIRA 3 (Brno, Czech Republic) instrument attached to dispersive X-ray spectrometry at an operating voltage from 8 to 15 kV. Overall, microstructural images were obtained by high-resolution transmission electron microscopy (HR-TEM, Talos F200S, Thermo Fisher Scientific, Eindhoven, The Netherlands). Powder X-ray diffraction (XRD) measurements were performed on a BRUKER-binary V3 powder diffractometer with Cu K_α_ radiation. Chemical oxidation states of the electrodeposited Pt were studied by X-ray photoelectron spectroscopy (XPS-Omicron nanotechnologies Instrument, GmbH, Taunusstein, Germany).

## 3. Results and Discussion

### 3.1. Pt Particle Electrochemically Deposited on A Carbon Substrate and Their Response to Varying Current Density

**Figure 1:**
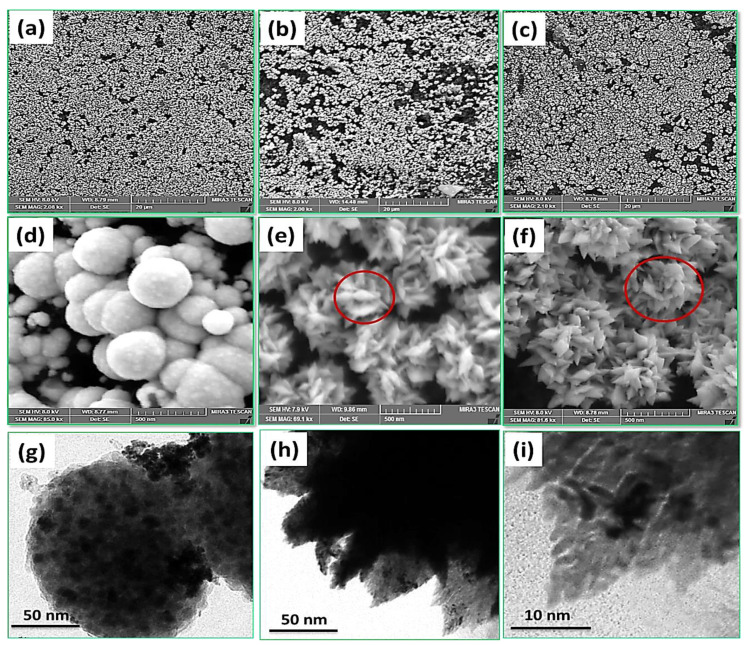
Low-magnification field emission scanning electron microscope (FE-SEM) micrographs (**a**–**c**) and high magnification (**d**–**f**) with various Pt deposition current densities of 1.5, 6, and 15 mA cm^−2^ and (**g**–**i**) high resolution transmission electron microscopy (HR-TEM) image with Pt deposition current densities of 1.5 and 6 mA cm^−2^. (**e**,**f**—circled region shows dentritic flower-like morphology)

Figure 1a–c shows the FE-SEM micrographs of Pt particles deposited at different current densities on the carbon substrate. It is clear from the low-magnification FE-SEM micrographs that the Pt particles are uniformly electrodeposited/grown on the carbon substrate under the chronopotentiometry electrodeposition condition current limits of 1.5, 6, and 15 mA cm^−2^, respectively. It is noteworthy that the platinum metal deposition was performed at room temperature, maintaining a bath pH of 1 at a stirring rate of ~800 rpm and a deposition time of 600 s, which were kept constant for all the samples. The high-magnification FE-SEM image of Figure 1d indicates the formation of a well-defined Pt sphere with an applied minimum current density of 1.5 mA cm^−2^. Over 150 particles confirm the shape distribution in the FE-SEM images, which appears to be a sphere. The above result suggests the effectiveness of using lower current density to control the shape of Pt and the formation of spheres. However, the Pt particle size varied from 100 to 300 nm on the carbon surface, as can be observed more clearly in Figure 1d. The particle size variation attributed to the self-limiting growth mechanism of Pt to a maximum to 300 nm, above which the Pt nucleation/growth happens on an adjacent site. As the current density limit increased from 1.5 to 3 and 6 mA cm^−2^, the sphere-shaped Pt nanoparticles slowly start to form a flower morphology (3 mA cm^−2^), as given in Figure S1. Comparatively increasing the current density to 6 mA cm^−2^, the above mixed Pt morphology entirely changed and formed flower with petals to look like a rose flower shape on a carbon substrate, as shown in Figure 1e. The experiment performed with a higher current density of 9 (Figure S1) and 15 mA cm^−2^ resulted in a homogeneous distribution of sharp petals with a core-rose flower shape from Figure 1f. It is clear from the above results that an increase in applied current density during electrodeposition results in an increasing growth process with more dendritic structures grown over the existing shapes.

Figure 1g shows the TEM micrograph of the Pt particles deposited at a current density of 1.5 mA cm^−2^, clearly indicating spherical morphology. This observation is in good agreement with the FE-SEM micrographs. These spherical Pt nanoparticles are found to be of high crystallinity and are formed by the group of smaller nanospheres with a size range of 1–3 nm. Relatively increasing Pt deposition current to 6 mA cm^−2^ resulted in the change from globular nanosphere structure to dendritic flower-like shape, as observed from Figure 1h. A higher-magnification micrograph reveals individual Pt has an oval shape with nanocrystalline nature, which is visible from Figure 1i. Besides, while increasing Pt deposition current density to 15 mA cm^−2^, many Pt particles are formed. However, during the experiment, while increasing the current density to more than 15 mA cm^−2^, the Pt deposition rate decreases on the carbon surface and form as crystal nature, and some Pt particle is found to be floating on the electrolyte. The chemical composition determined by Energy Dispersive X-RayAnalyzer (EDX) specifies that Pt particles are deposited on the carbon, and no other impurities are observed (Figure S2).

**Figure 2:**
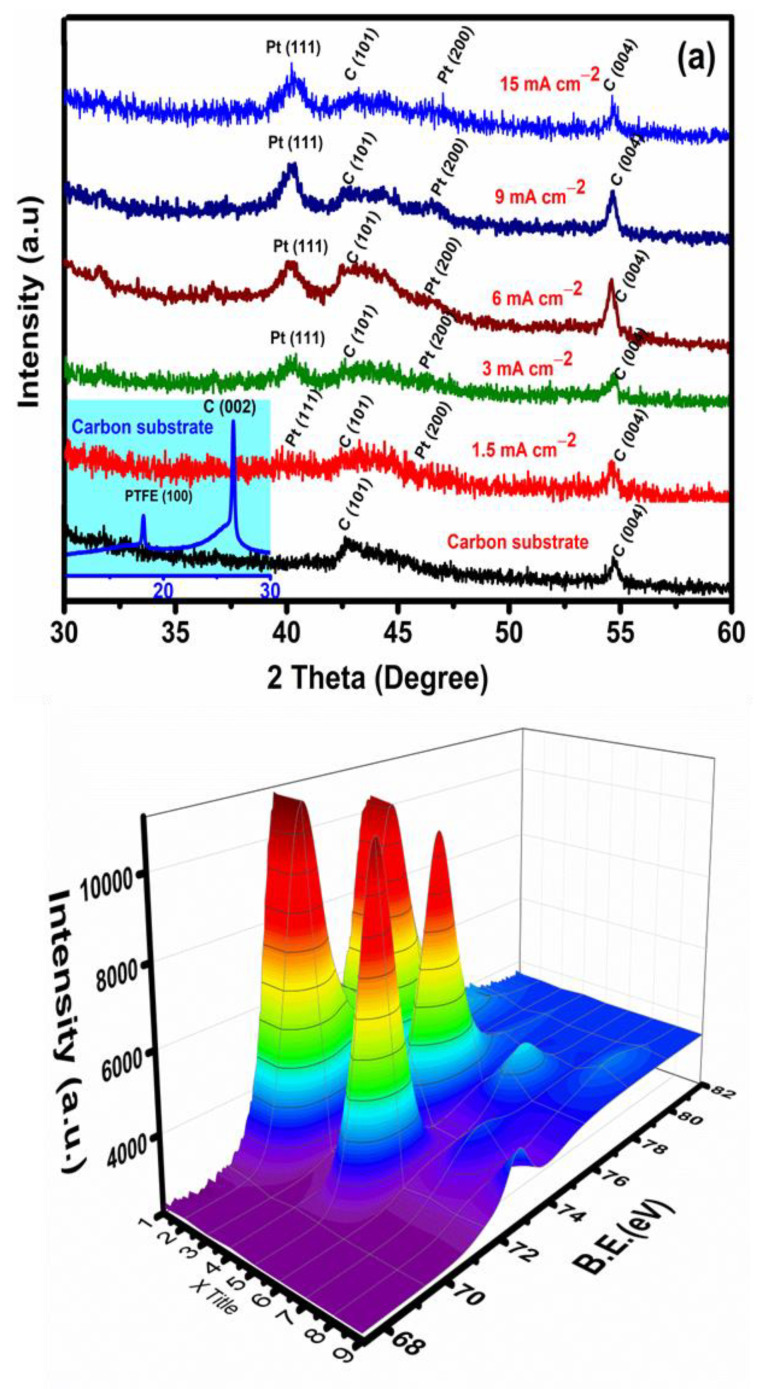
(**a**) Typical X-ray diffraction (XRD) pattern with Pt deposition of different current densities on a carbon substrate. (Note: The expended region between 10° and 30° indicated two different peaks due to carbon and PTFE observed for all samples). (**b**) 3D view XPS spectrum of Pt 4f binding energy orbital.

The multi-segmented Pt crystal structure with different shapes synthesized over carbon electrodes is characterized by X-ray diffraction. Figure 2a shows that all samples have identical XRD profiles. The XRD pattern of Pt electrodeposited at different current densities indicates that the sphere and flower correspond to the face center cubic phase, which is in good agreement with JCPDS # 87-0647. The peaks at 39.8°, 46.2°, and 67.8° attributes to the (111), (200), and (220) diffraction lines, which confirm that Pt is in the metallic state. In the present work, while increasing Pt deposition current density, the rate of Pt deposition on carbon surface and crystallinity also improved. The XRD results indicate that the growth formation and crystallinity increase with increasing Pt deposition current density. The expended region between 10° and 30° is given in Figure 2a inset. The sharp peak at 18.1° and 26.4° corresponds to (100) and (002) facets of PTFE and graphitic carbon observed from carbon substrate and consistently reflected for all samples.

Furthermore, to analyze/examine Pt nanoparticles surface oxidation states, XPS studies were conducted. XPS survey spectrum for Pt deposited on carbon substrate at a current density of 6 mA cm^−2^ is shown in Figure S3, which reveal predominant peaks of oxygen, carbon and platinum, and no other impurities are detected. Moreover, the de-convoluted Pt 4f peak in Figure 2b shows three doublet binding energy peaks. The Pt 4f core level has a sharp peak at 71.2 and 74.5 eV, which is associated with Pt 4f 7/2 and 4f 5/2, representing that Pt has a highly metallic state. Similarly, an interim and minor peak at 72.4 and 76.3 eV and 73.4 and 78.2 eV predicted Pt to be in (+2) and (+4) oxidation states.

### 3.2. Influence of Time on Pt Particle Deposited on the Carbon Substrate

**Figure 3:**
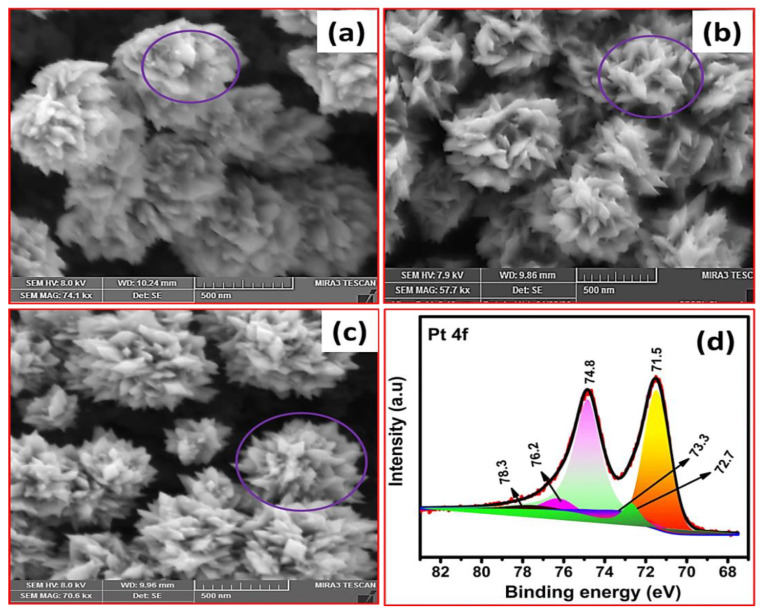
(**a**–**c**) FE-SEM micrographs for Pt structure deposited on a carbon substrate with the effect of time (300, 600, and 900 s) and (**d**) XPS spectrum of Pt 4f binding energy orbital with the effect of 900 s. (**a**–**c**—circled region shows flower-like morphology)

Figure 3 shows FE-SEM images of Pt materials grown onto a carbon substrate by electrochemical deposition route in relation to time. For this specific purpose, the current density was kept at 6 mA cm^−2^, the temperature of 28 °C, and pH of 1 maintained at a stirring rate of ~800 rpm. All these parameters were kept constant with time as the variable, and the Pt flower morphological parameters comprising the deposition rate and growth formation were investigated. It can be observed that all Pt film appeared of distinctive dendritic nature with a flower shape grown perpendicularly on a carbon substrate. The lower magnification images show that the deposition rate strongly increases with increasing deposition time. Pt structures almost covered the entire carbon substrate due to increased deposition duration from 300, 600, and 900 s.

Figure 3a reveals during Pt deposition for 300 s, and it can be seen that Pt flower bud growth starts and petals are open slightly. Similarly, with increasing Pt deposition time of 600 s, Pt flower grown on carbon surface and petals are more distinctively visible, as shown in Figure 3b. While increasing deposition time to 900 s, more petals continue to grow on the substrate and show a sharp core-flower shape. It can be observed that the length-growth rate and petals formation intensely increase from 300 to 900 s duration time. Figure 3d shows XPS spectra of electrodeposited Pt on carbon substrate for the duration of 900 s. The de-convoluted XPS spectra of Pt 4f binding energy peaks reveal that Pt has a metallic state acceding with different literature reports [2,18,26]. Similarly, XRD results also indicate that Pt has a face-center-cubic with the metallic state, as shown in Figure S4.

### 3.3. Effect of Temperature on Pt Structure Fabricated on a Carbon Substrate

**Figure 4:**
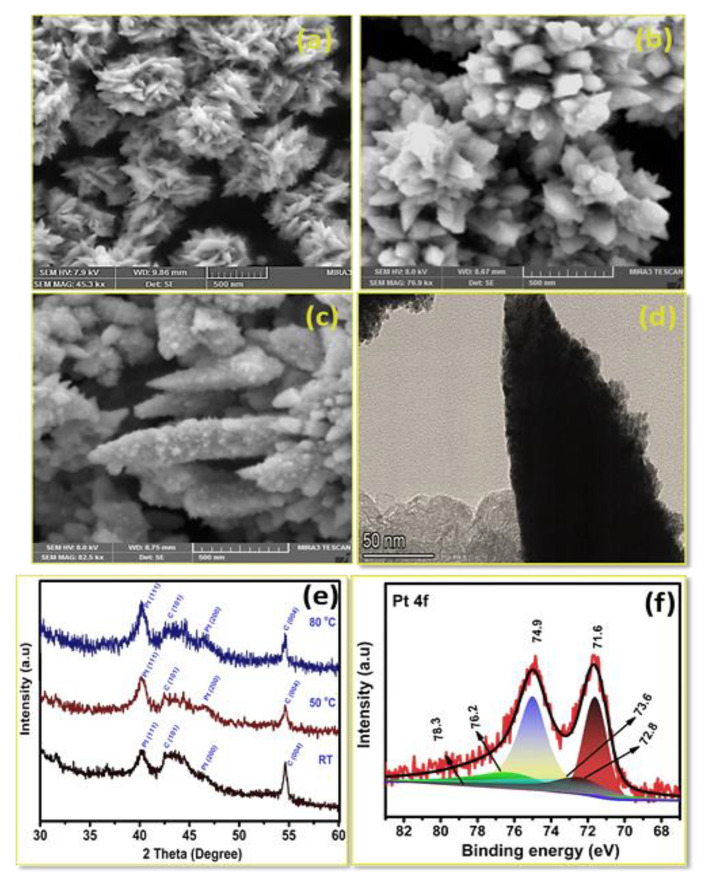
FE-SEM micrographs of Pt deposited on carbon substrate with effect of bath temperature; (**a**) room temperature, (**b**) 50 °C, (**c**) 80 °C, (**d**) HR-TEM image of Pt deposition (80 °C), (**e**) XRD pattern, and (**f**) XPS Pt 4f spectrum of Pt deposition at (80 °C).

The effect of temperature substantially affects the appearance of surface morphology and crystallinity of the deposited metal nanoparticles. To analyze the precise effect of temperature, all other electrodeposition parameters were kept constant. During the Pt deposition process, a current density of 6 mA cm^−2^, deposition time of 600 s, bath pH of 1, and stirring rate of ~800 rpm was maintained for all the temperatures. Figure 4 displays the FE-SEM images of Pt grown on a carbon substrate with varying bath temperatures. In the present work, bath temperature is increased from room temperature to 80 °C. Pt deposited at room temperature reveal a rose-flower shape, and petals are more distinctively visible/open, as shown in Figure 4a. However, while increasing the bath temperature to 50 °C, a change of the Pt petals morphology to rods is observed from Figure 4b. Further increase in electrolyte bath temperature to 80 °C resulted in increased Pt rods size with sharp crystal growth, as shown in Figure 4c. The increased electrolyte bath temperature is believed to decrease the electrolyte viscosity, thereby replenishing the double layer quite faster [27]. It also increases solubility, leading to increased electrolyte conductivity resulting in the growth process gradually. According to Sunil Kumar et al. the deposition process at a high-bath temperature usually decreases the adsorption of hydrogen on the deposits and reduces stress [28].

Further, the HR-TEM image reveals that Pt has a nanorod shape with sharp edges and rough surface, observed in Pt deposited on a carbon substrate at a temperature of 80 °C, as shown in Figure 4d. In addition to that, an increase in bath temperature from room temperature to 80 °C resulted in increased grain size of Pt metal particles as observed from XRD measurement (Figure 4e). Moreover, XPS of Pt deposited at a temperature of 80 °C also clearly reveals that Pt is in a metallic state (Figure 4f).

The above results indicate that the formulated electrolyte ion present has a certain zigzag Brownian motion at room temperature. This random motion of ions may collide with other ions, resulting in ionic direction changes in an applied electric field. At room temperature and an applied current density of 6 mA cm^−2^, the cell voltage was low in the initial stage (1.8 V) and started increasing at a quite faster rate (2.2 V) during the initial 50 s, and once Pt metal on a carbon substrate was saturated, the decrement in voltage was relatively slow (2.1 V). The reason is that Pt growth continues faster at the initial stage on carbon substrate and petals are more open during the optimum time and temperature, which is in good agreement with the effect of time study as given in Figure 3a. Further, the electrodeposition was studied at 50 °C, and the electrodeposition was commenced as discussed above, and the applied current density (6 mA cm^−2^) was maintained.

As discussed earlier, during Pt deposition at room temperature, the initial voltage was high and started to decrease gradually with time. However, at 50 °C, the initial voltage is higher in the initial stage (1.82 V) and a bit lower after 100 s (1.7 V) and slowly saturated, however the overall voltage lesser than the room temperature experiment. The reason may be, at a higher temperature, there is more Brownian motion in the formulated electrolyte, and the formation of branches change look like a rod shape and deposition also enhanced as compared to room temperature. Experiments carried out for Pt deposition at 80 °C also exhibit almost similar trends and the tendency of reducing cell voltage of 1.56 V. It is clear from the FE-SEM micrographs of electrodeposited Pt at a higher temperature that growth patterns are highly dense compared to that of Pt electrodeposited at a lower temperature, as shown in Figure 4c. In addition, the branch size also increased with relatively thicker and wider stems, as evident from the FE-SEM micrographs. This may be due to increased bath temperature; the Influence of the ions random Brownian motion has increased, resulting in thicker branches and denser Pt deposit.

During Pt deposition at room temperature, Pt grows into distinct branches, and the formation of the rose flower shape is seen. While increasing bath temperature to 50 °C, branches tend to grow faster at the later stage. Further, the surface morphology’s overall appearance diverges from the one obtained at room temperature, where the rods were formed and more of an open structure. In addition, while increasing the temperature to 80 °C, it appeared that surface morphology had become relatively thicker, with a rough surface and sharp edges visible, as seen from Figure 4c,d, compared to low temperature (Figure 4b). This formation is due to increasing Brownian motion of higher magnitude at a higher temperature, leading to an excess of agitation and thermal drifting of ions in the electrolytic solution.

### 3.4. Pt Structure on A Carbon Substrate with the Influence of pH

**Figure 5:**
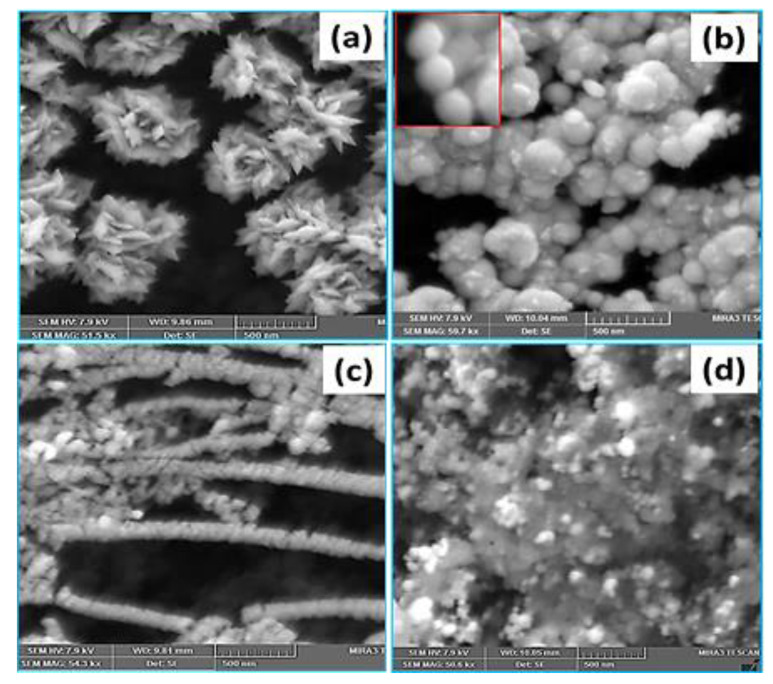
FE-SEM images of Pt deposited with influence of pH: (**a**) pH 1, (**b**) pH 3, (**c**) pH 7, and (**d**) pH 10. (**b**—inset shows pellet-shaped globular morphology of deposited Pt)

The Pt particle deposition and growth kinetics during electrochemical deposition from electrolytes of different pH and overall mass rates are alleged to play a specifically important role in forming different shapes. Figure 5a–d shows the surface morphology of Pt metal particle electrodeposited at different electrolyte pH. For this study, deposition parameters other than pH were kept constant in all cases, viz., the current density (6 mA cm^−2^), bath kept at temperature, deposition time of 600 s and stirring rate of ~800 rpm was maintained.

**Figure 6:**
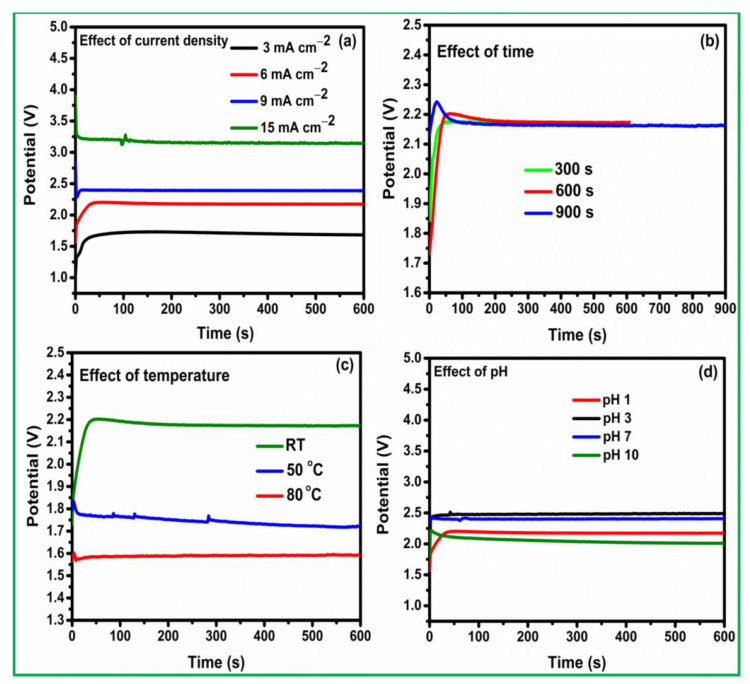
Pt deposition on a carbon surface via the (**a**) effect of current density, (**b**) effect of time (constant current density), (**c**) effect of temperature, and (**d**) effect of pH with constant current density (6 mA cm^−2^).

FE-SEM micrographs in Figure 5a for electrodeposited Pt at a lower pH of formulated electrolyte reveals flower-like (rose) morphology of Pt. The Pt metal films are very thin, mainly caused by the high hydrogen evolution during electrodeposition at low pH values with respect to current density. With increasing the pH to value 3, the Pt film’s overall surface is observed to be smooth and uniform compared to lower pH, which may be due to a smaller amount of hydrogen evolution while increasing pH. Similarly, the surface shape also changes from flower morphology to pellet shape, which is observed from Figure 5b. The Pt film’s surface significantly increased by increasing formulated pH up to 7 using NaOH, resulting in the chain-like shape, as shown in Figure 5c. Further, when the pH is increased to 10, metal hydroxides such as PtOH form and begin precipitating in the electrolyte. The surface morphology changed and formed the agglomeration/Nodular structure of Pt. In addition to that, a higher pH of formulated electrolytes favors the formation and absorption of metal hydroxides and depresses the freshly deposited Pt metal. The electrochemical deposition curves indicating the Influence of current density, time, temperature, and pH for Pt deposition on carbon are given in Figure 6. The change of Pt morphology in relation to these parameters is discussed in detail above.

An optimized electrode obtained via electrochemical deposition was subjected to real-time PEFC testing for its durability. The electrochemically deposited Pt on carbon obtained with the applied current density of 6 mA cm^−2^ was used as an anode with metal loading of 18 µg cm^−2^, and commercially available Pt/C metal loading of 50 µg cm^−2^ was used as a cathode. (Note: An optimum amount of 40 wt % of commercial Pt/C was wet with de-ionized water followed by the addition of 5 wt % of Nafion ionomer and IPA solution and then sonicated for 45 min. The above admixture was coated on GDL (DC-35 commercially available carbon electrode). For comparative studies, commercially available Pt/C was used as an anode (50 µg cm^−2^ Pt) and a cathode (50 µg cm^−2^), respectively. Nafion-HP membrane was then placed between the above two electrodes to design a membrane electrode assembly (MEA) by hot-press with a compaction pressure of 20 kg cm^−2^ for 2 min. PEFC test was performed in a cell fixture of 25 cm^2^ (Fuel cell technologies). The steady-state performance and stability test was carried out under the dry gas condition with an operating temperature of 40 °C using Birode Instruments.

**Figure 7:**
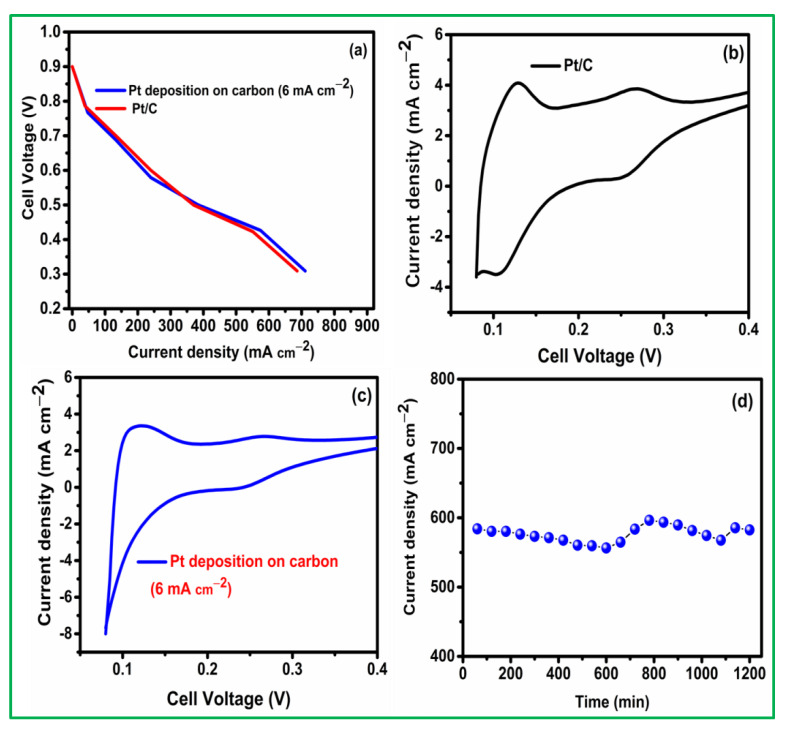
(**a**) Fuel cell performance, (**b**,**c**) H_2_ adsorption and desorption behaviour of Pt/C and Pt deposited on carbon (6 mA cm^−2)^, and (**d**) stability test at 0.4 V for Pt deposited on carbon (6 mA cm^−2^).

Figure 7a shows steady-state data for Pt/C and Pt particle electrochemically deposited on carbon. Pt deposited on carbon with an optimum current density of 6 mA cm^−2^ shows a superior current density of 560 mA cm^−2^ at 0.4 V on par with the commercial Pt/C. Similarly, Figure 7b,c, shows Pt deposition on carbon (6 mA cm^−2^) and Pt/C have better H_2_ adsorption and desorption behaviour. Similarly, under dry gas operating condition, Pt deposited on carbon shows higher stability of 550 mA cm^−2^ at 0.4 V even after 1200 min, as shown in Figure 7d. Ye et al. prepared PtRuIr nanoclusters by selectively pulsing electrodeposition method and reported that the optimum concentration of Ir in the PtRu bifunctional electrocatalyst resulted in a higher current density of 150 mA cm^2^ at 0.4 V under humidified H_2_:O_2_ configuration [29].

Similarly, Yang et al. prepared PtNi alloy octahedral nanoparticles electrochemically deposited on carbon substrate via pulse-like hydrothermal method. The authors reported that the effect of temperature on the pulse deposition method could result in speeding up the growth process, and the final Pt-Ni alloy morphology was observed to be different due to the different growth times and temperatures. Besides, during oxygen reduction reaction Pt-Ni octahedral alloy exhibited a 50 mV positive shift of the half-wave potential compared to commercial Pt/C [30]. In addition, Liu et al. group also studied the mechanism and kinetic study of the pulse electrodeposition process of Pt on carbon. During pulse current electrodeposition, larger current densities are suitable for the Pt deposition process, which may be leading to larger cathode overpotentials, resulting in increased nucleation rate and beneficial to form smaller Pt metal particles. Similarly, introducing proper additives is also useful for forming various morphology and improving fuel cell performance progressively [31,32].

The present work reveals that the direct current deposition process with lower metal loading of Pt deposited on carbon shows superior fuel cell performance and the peak power density of 250 mW cm^−2^ at 0.4 V. Moreover, during dry gas operation, it shows higher stability of 550 mA cm^−2^ at 0.4 V even after 1200 min. While initial fuel cell studies with the electrochemically deposited Pt on carbon are promising, the other important parameter for the Pt morphology with time, temperature, and pH as anode and cathode electrocatalyst needs to be studied in detail. Further studies are in progress.

## 4. Conclusions

In conclusion, different shapes of surface morphologies of Pt metal particles are fabricated by a one-step electrochemical approach. The applied current density is the most critical factor to change the surface morphology from sphere to core-rose flower shape with a desired size and crystallinity growth. The formation of the Pt flower growth mechanism was also successfully investigated at varying times. In addition, instantaneous nucleation was found by varying temperature and pH to have a significant impact on surface morphology and crystallinity. Electrochemically deposited Pt on carbon substrate with optimum current density shows excellent fuel cell stability under dry gas condition. The above results reveal that electrochemical deposition of Pt materials with different morphology opens a wide application in sustainable energy.

## Data Availability

Data sharing not available.

## Supplementary Materials

The following are available online at https://www.mdpi.com/article/10.3390/ma14092330/s1, Figure S1. FE-SEM image for Pt deposition on carbon substrate with a current density of 3 and 9 mA cm^−2^, Figure S2. EDX measurement of Pt nanoparticles is deposited on a carbon substrate with different current density, Figure S3. XPS survey spectrum for Pt deposited on a carbon substrate at a current density of 6 mA cm^−2^, Figure S4. XRD pattern with Influence of time on Pt nanoparticle deposited on carbon substrate.
