# Supplementary material for: Insight towards Nucleation Mechanism and Change in Morphology for Nanostructured Platinum Thin Film Directly Grown on Carbon Substrate via Electrochemical Deposition
Source: Materials (Basel). 2021 Apr 30;14(9):2330. doi: 10.3390/ma14092330 (PMC8124617; doi:10.3390/ma14092330)
Supplement: Supplementary file 1 [file materials-14-02330-s001.zip › materials-1183404-supplementary.pdf]

materials

Supporting Information

Insight towards Nucleation Mechanism and Change in Morphology for Nanostructured Platinum Thin Film Directly Grown on Carbon Substrate via Electrochemical Deposition

Prabhakaran Dhanasekaran [1,*], Swaminathan Rajavarman [2], Sivasuriyanarayanan Vinod Selvaganesh [3] and Santoshkumar Dattatray Bhat [1,*]

[1] CSIR-Central Electrochemical Research Institute (CECRI), CSIR-Madras Complex, Tamil Nadu 630003, India
[2] Centre for Nanoscience and Nanotechnology, University of Madras, Tamil Nadu 600085, India; srajavarman11@gmail.com
[3] Indian Institute of Technology, Madras, Chennai, Tamil Nadu 600036, India; svsganesh.dr@gmail.com
* Correspondence: dhanascient@gmail.com (P.D.); sdbhat@cecri.res.in (S.D.B); Tel./Fax: +91-44-22542456 (S.D.B.)

Supporting Information

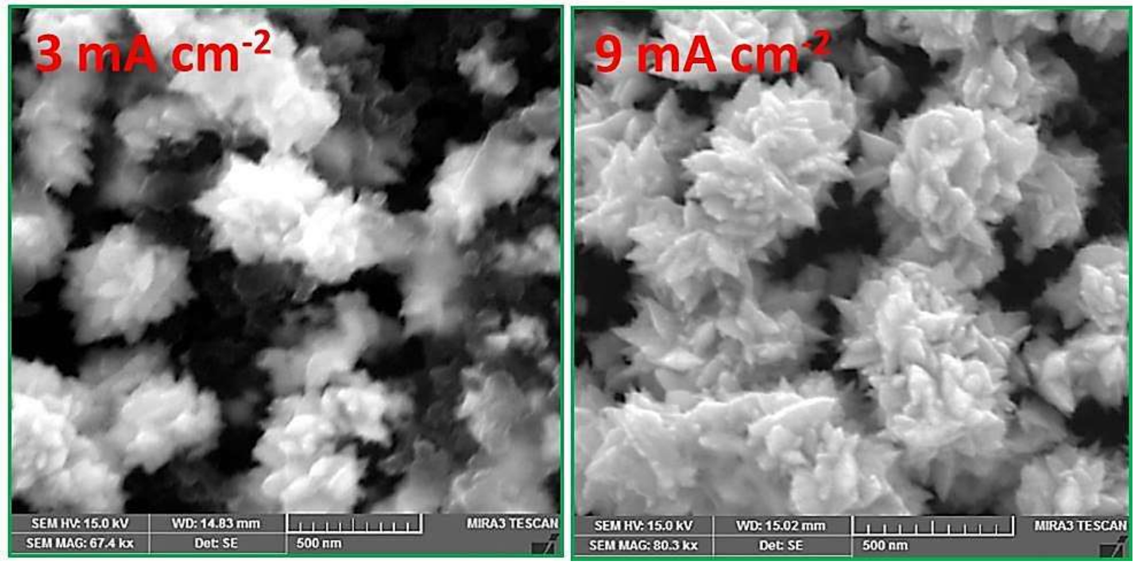

Figure S1. FE-SEM image for Pt deposition on carbon substrate with a current density of 3 and 9 mA cm^{-2}.

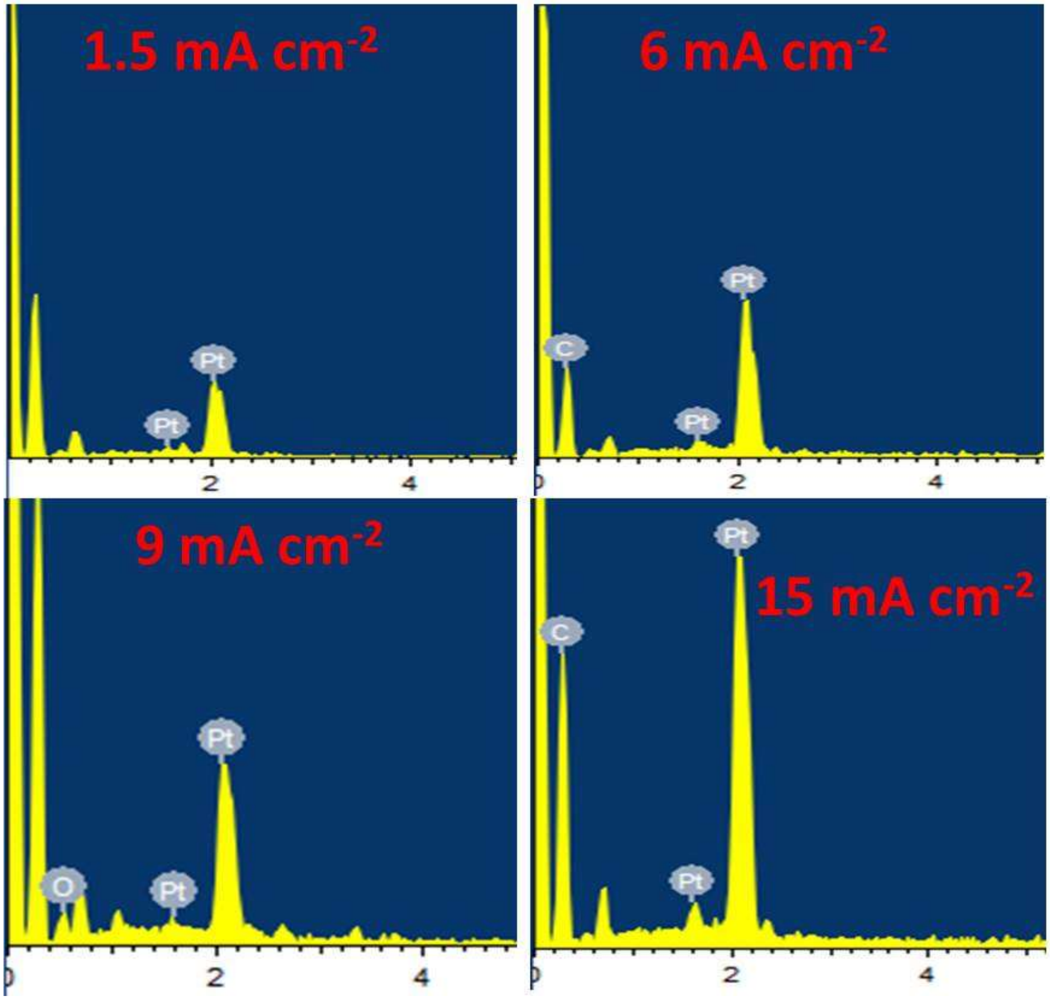

Figure S2. EDX measurement of Pt nanoparticles is deposited on a carbon substrate with different current density.

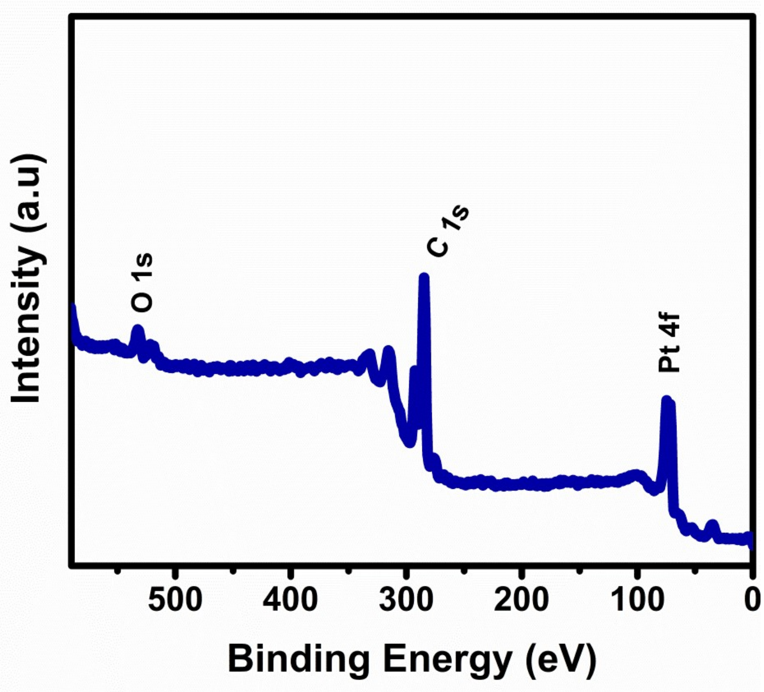

Figure S3. XPS survey spectrum for Pt deposited on a carbon substrate at a current density of 6 mA cm^{-2}.

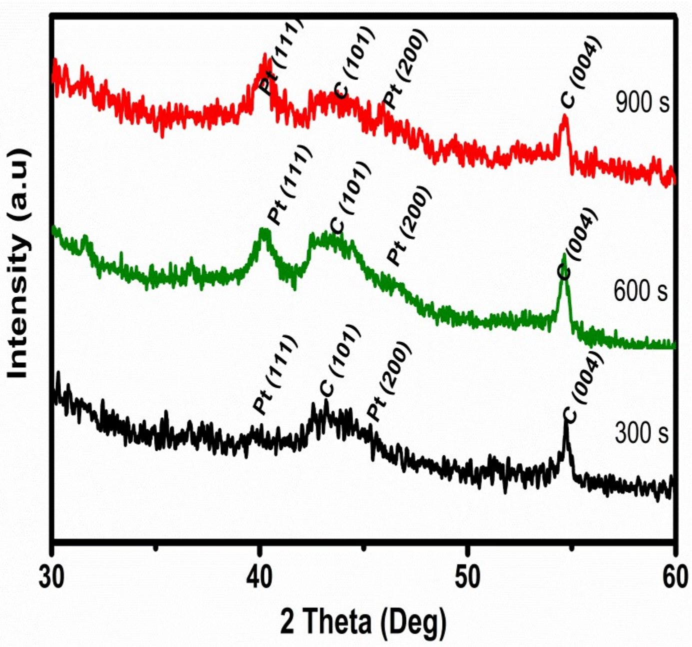

Figure S4 XRD pattern with Influence of time on Pt nanoparticle deposited on carbon substrate.
